# The DBP Phenotype Gc-1f/Gc-1f Is Associated with Reduced Risk of Cancer. The Tromsø Study

**DOI:** 10.1371/journal.pone.0126359

**Published:** 2015-05-18

**Authors:** Rolf Jorde, Henrik Schirmer, Tom Wilsgaard, Ellisiv Bøgeberg Mathiesen, Inger Njølstad, Maja-Lisa Løchen, Ragnar Martin Joakimsen, Guri Grimnes

**Affiliations:** 1 Tromsø Endocrine Research Group, Department of Clinical Medicine, UiT The Arctic University of Norway, Tromsø, Norway; 2 Division of Internal Medicine, University Hospital of North Norway, Tromsø, Norway; 3 The School of Population Health, University of Auckland, Auckland, New Zealand; 4 Department of Clinical Medicine, UiT The Arctic University of Norway, Tromsø, Norway; 5 Epidemiology of Chronic Diseases Research Group, Department of Community Medicine, UiT The Arctic University of Norway, Tromsø, Norway; 6 Brain and Circulation Research Group, Department of Clinical Medicine, UiT The Arctic University of Norway, Tromsø, Norway; 7 Department of Neurology and Neurophysiology, University Hospital of North Norway, Tromsø, Norway; University of Alabama at Birmingham, UNITED STATES

## Abstract

**Background and Objective:**

In addition to its role as a transport protein, the vitamin D binding protein (DBP) may also affect lipid metabolism, inflammation and carcinogenesis. There are three common variants of the DBP, Gc1s (1s), Gc1f (1f), Gc2 (2) that result in six common phenotypes (1s/1s, 1s/1f, 1s/2, 1f/1f, 1f/2, and 2/2). These phenotypes can be identified by genotyping for the two single nucleotide polymorphisms rs7041 and rs4588 in the GC gene. The DBP variants have different binding coefficients for the vitamin D metabolites, and accordingly there may be important relations between DBP phenotypes and health.

**Methods:**

DNA was prepared from subjects who participated in the fourth survey of the Tromsø Study in 1994-1995 and who were registered with the endpoints myocardial infarction (MI), type 2 diabetes (T2DM), cancer or death as well as a randomly selected control group. The endpoint registers were complete up to 2010- 2013. Genotyping was performed for rs7041 and rs4588 and serum 25-hydroxyvitamin D (25(OH)D) was measured.

**Results:**

Genotyping for rs7041 and rs4588 was performed successfully in 11 704 subjects. Among these, 1660 were registered with incident MI, 958 with T2DM, 2410 with cancer and 4318 had died. Subjects with the DBP phenotype 1f/1f had 23 – 26 % reduced risk of incident cancer compared to the 1s/1s and 2/2 phenotypes (P < 0.02, Cox regression with gender as covariate). Differences in serum 25(OH)D levels could not explain the apparent cancer protective effect of the DBP variant 1f. In addition to cancer and 25(OH)D, there were significant associations between DBP phenotype and body height, hip circumference and serum calcium.

**Conclusion:**

There are important biological differences between the common DBP phenotypes. If the relation between the DBP variant 1f and cancer is confirmed in other studies, determination of DBP phenotype may have clinical importance.

## Introduction

Vitamin D binding protein (DBP) is an α_2_-globulin that functions as a carrier protein for vitamin D and its metabolites. It was originally known as the Group-specific component (Gc-globulin) and is encoded by the *GC* gene on chromosome 4q12-q13 [1]. In the circulation about 85%- 90% of 25-hydroxyvitamin D (25(OH)D), the metabolite used to evaluate a subject´s vitamin D status, and 1,25-dihydroxyvitamin D (1,25(OH)_2_D), the active form of vitamin D, are bound to DBP. A considerable amount is also bound to albumin and less than 1% of 25(OH)D and 1,25(OH)_2_D circulate in the bloodstream freely [2]. There are a number of variants of DBP that can be distinguished by their electrophoretic migration pattern of which three variants Gc1f (1f), Gc1s (1s) and Gc2 (2) are common [3]. These three variants are caused by two single nucleotide polymorphisms (SNPs) in the *GC* gene, rs7041 and rs4588, and result in six common DBP phenotypes (1s/1s, 1s/1f, 1s/2, 1f/1f, 1f/2, and 2/2) [4]. The three DBP variants have different affinity for 25(OH)D and 1,25(OH)_2_D, and accordingly, the amount of free vitamin D metabolite depends not only on amounts of DBP and albumin, but also on DBP phenotype [5].

In addition to its importance for bone health, vitamin D has been ascribed a number of other functions and low serum 25(OH)D is associated with diseases like cancer, cardiovascular disease (CVD) and diabetes [6]. There has recently been an interest in whether the free form of 25(OH)D is a better indicator of vitamin D status than total 25(OH)D, which is usually measured [4]. Thus, there are reports that the free or bioavailable form of 25(OH)D correlates better with bone density than total 25(OH)D [7, 8], and if this also applies to other effects of vitamin D, then DBP phenotype would attain an even more important role in research on vitamin D related health.

DBP also has functions outside vitamin D metabolism. DBP is a carrier of saturated and unsaturated free fatty acids and may therefore be involved in lipid metabolism [9]; DBP binds actin with high affinity and is vital in preventing actin polymerization when actin is released during tissue damage [10]; DBP plays a part in the inflammation response as it enhances the effects of complement derived C5a on neutrophil and macrophage chemotaxis [11]; and finally, a deglycosylated form of DBP may act as a macrophage activator (DBP-MAF) which may affect not only phagocytic and tumoricidal activity but also osteoclast differentiation [12, 13]. To a large degree these effects are influenced not only by amount of DBP, but also by DBP variants [14]. Differences in the DBP phenotypes may therefore be important for health through several mechanisms, and it is no surprise that there are a number of publications on relations between DBP and various diseases; however, the results are equivocal [15, 16].

In Tromsø, a town in Northern Norway, repeated population-based health surveys have been conducted at 6–7 years intervals since 1974 [17]. In the fourth survey performed in 1994–1995, blood samples for preparation of DNA were collected in close to 27 000 subjects and genotyping for DBP performed in 11 704 subjects. The participants have been followed-up with registration of incident myocardial infarction (MI), type 2 diabetes (T2DM), cancer and death, and we therefore had the opportunity to test if DBP genotypes are related to these hard endpoints as well as to anthropometric measures and CVD risk factors in a large cohort.

## Materials and Methods

### The Tromsø Study

The Tromsø Study is a longitudinal population-based health study conducted by UiT the Arctic University of Norway. The participants are inhabitants of the municipality of Tromsø which is the largest city in Northern Norway situated at 69° N with a current population of 72 000 subjects. The fourth survey was performed in 1994–1995 (“Tromsø 4”), and 27 158 subjects attended. The design of the Tromsø Study has been described in detail by Jacobsen et al. [18].

### Measurements

In Tromsø 4 the participants filled in questionnaires on medical history including smoking habits and antihypertensive medication. Blood pressure, height and weight were measured in all and hip and waist circumference in a subgroup; non-fasting blood samples were analysed consecutively for serum calcium, total cholesterol, high-density lipoprotein (HDL) cholesterol, and triglycerides in all subjects; and HbA_1c_ and serum parathyroid hormone (PTH) in subgroups as previously described [19].

Sera were stored at -70°C, and after a median storage time of 13 years, thawed in March 2008 and analysed for 25(OH)D in a subgroup using an automated clinical chemistry analyser (Modular E170, Roche Diagnostics, Mannheim, Germany). This assay overestimates serum 25(OH)D in smokers [20], and the serum 25(OH)D levels are therefore shown for non-smokers only.

DNA was prepared from blood clots stored at the national CONOR (Cohort of Norway) biobank, located at the HUNT/NTNU biobank in Levanger. To determine the three common DBP variants (1f, 1s and 2), we genotyped the subjects for rs7041 and rs4588. The genotyping was performed by KBioscience (http://www.kbioscience.co.uk) using KBioscience Competitive Allele-Specific PCR genotyping system [19].

### Selection of subjects and definition of endpoints

The selection of subjects for the genetic studies has been described in detail previously [19]. In short, the subjects were primarily selected for evaluation of genetic polymorphisms and clinical endpoints for which there are several registers in the Tromsø Study including MI, T2DM, stroke, hip and radial fractures; in addition information on cancer and death is available from national registries. As all of these endpoints were of potential interest regarding genetic polymorphisms, and limited funding did not allow genetic analyses of the entire Tromsø Study cohort, a case-cohort design was used with randomly selected controls from the entire cohort who attended Tromsø 4 [21]. Accordingly, genotyping was performed in all subjects included in the endpoint registers as well as the randomly selected controls.

The definitions of the clinical endpoints MI, T2DM, cancer and death endpoints have been described in detail [19]. In short, possible cases of T2DM and MI were identified by linkage of the fourth survey participant list to the University Hospital of North Norway digital discharge diagnosis registry. The hospital medical record was then retrieved for case validation. In addition, a systematic manual and electronic search on all participants registered with cardio-vascular diagnosis codes were performed. The T2DM and MI endpoints were included till the end of 2011, but completely updated till the end of 2010. Information on cancer incidence and cancer location was retrieved from the Cancer Registry of Norway updated till the end of 2010. Information on death was obtained from the Causes of Death Registry, and information on moving out of the Tromsø area and emigration from Norway was obtained from the Norwegian Registry of Vital Statistics updated till 18 January 2013.

### Statistical analyses

Distribution of the continuous variables was evaluated for skewness and kurtosis and visual inspection of histograms and found normal except for triglycerides and HbA_1c_, which were normalized by log transformation before use in the statistical analyses. Assuming that differences between phenotypes would be most easily detected between the three homozygote variants (1s/1s, 1f/1f, 2/2), these were primarily compared using ANOVA with Bonferroni correction for multiple testing. Trends across phenotypes (e.g. two homozygote phenotypes and their corresponding heterozygote phenotype) were evaluated with linear regression with gender as covariate.

The relations between the endpoints (MI, T2DM, cancer, death) and DBP phenotype were initially evaluated with the Chi-square test, and subsequently with Cox regression with gender as covariate. The observation time was set from date of attendance in Tromsø 4, and accordingly only incident endpoints were included. Those with endpoints before Tromsø 4 were excluded from the analyses. For these analyses the predefined control cohort was used as controls for the subjects with a specific endpoint (cases). Since this control cohort was randomly selected from the entire Tromsø 4 cohort, it also included a considerable number of subjects with one or more endpoints. When analysing a specific endpoint, subjects in the control cohort with that specific endpoint were moved to the case group (which only included subjects with that specific endpoint). Therefore, the size of the control cohort varied depending on endpoint in question.

The data are shown as mean ± SD. All tests were done two-sided, and a P-value < 0.05 was considered statistically significant.

### Ethics

The Regional Committee for Medical and Health Research Ethics, REK Nord, approved the study (REK Nord, reference 2010/2913-4). Only participants with valid written informed consent were included. The study was registered at ClinicalTrials.gov (NCT01395303).

## Results

Genotyping was performed in 12 029 subjects, successfully for rs7041 in 11 834 subjects and for rs4588 in 11 762, enabling phenotyping for DBP in 11 704 subjects. The characteristics of the subjects in relation to DBP phenotype are shown in Table 1.

**Table 1:** Characteristics of the 11 704 subjects in relation to DBP phenotypes.

	DBP phenotypes					
	1s/1s	1s/1f	1s/2	1f/1f	1f/2	2/2
**N (%)**	3621 (30.9)	2456 (21.0)	3315 (28.3)	510 (4.4)	1104 (9.4)	698 (6.0)
**Females (%)**	1963 (54.2)	1351 (55.0)	1863 (56.2)	296 (58.0)	593 (53.7)	371 (53.2)
**Smokers (%)**	1267 (35.0)	840 (34.2)	1111 (33.5)	166 (32.5)	351 (31.8)	228 (32.7)
**Age (years)**	57.5 ± 13.6	57.4 ± 13.6	58.2 ± 13.6	57.5 ± 14.2	58.6 ± 13.5	58.2 ± 12.9
**Users of BP medication (%)**	418 (11.5)	309 (12.6)	374 (11.3)	59 (11.6)	132 (12.0)	75 (10.7)
**Systolic BP** * **(mmHg)**	140.5 ± 22.0	141.3 ± 22.1	140.5 ± 22.1	140.1 ±22.8	140.8 ± 22.2	141.5 ± 22.5
**Diastolic BP (mmHg)** *	81.2 ± 12.7	81.6 ± 12.8	81.0 ± 12.9	81.0 ± 12.4	81.6 ± 12.8	81.4 ± 12.5
**Body height (cm)**	168.4 ± 9.7	167.6 ± 9.5	167.9 ± 9.5	166.9 ± 10.0†	167.9 ± 9.7	168.2 ± 9.2
**Body weight (kg)**	73.5 ± 14.0	73.3 ± 14.3	72.5 ± 13.6	72.7 ± 14.6	73.6 ± 13.4	73.3 ± 13.2
**BMI (kg/m^2^)**	25.9 ± 4.1	26.0 ± 4.2	25.6 ± 4.0	26.0 ± 4.2	26.1 ± 4.0	25.8 ± 3.9
**Total cholesterol (mmol/L)**	6.51 ± 1.34	6.57 ± 1.31	6.56 ± 1.32	6.61 ± 1.29	6.59 ± 1.31	6.53 ± 1.32
**HDL-cholesterol (mmol/L)**	1.53 ± 0.43	1.52 ± 0.43	1.55 ± 0.44	1.51 ± 0.43	1.51 ± 0.43	1.52 ± 0.42
**Triglycerides (mmol/L)**	1.65 ± 1.07	1.07 ± 1.11	1.64 ± 1.06	1.65 ± 1.06	1.67 ± 1.06	1.67 ± 1.08
**Serum calcium (mmol/L)**	2.38 ± 0.10††	2.38 ± 0.11	2.38 ± 0.10	2.40 ± 0.10	2.38 ± 0.10	2.39 ± 0.10
**Circumference hip (cm)** **	103.5 ± 8.1	103.2 ± 8.0	102.9 ± 7.7	104.6 ± 8.9†††	103.8 ± 7.4	102.9 ± 6.7
**Waist/hip (ratio)** **	0.87 ± 0.08	0.88 ± 0.09	0.87 ± 0.08	0.86 ± 0.08†††	0.87± 0.08	0.88 ± 0.08
**Circumference waist (cm)** **	90.2 ± 11.7	90.5 ± 11.6	89.2 ± 11.3	89.8 ± 11.5	90.5 ± 10.9	90.2 ± 11.2
**HbA_1c_ (%)** ***	5.47 ± 0.74	5.46 ± 0.66	5.44 ± 0.62	5.43 ± 0.67	5.47 ± 0.60	5.43 ± 0.56
**Serum PTH (pmol/L)** ****	2.73 ± 1.42	2.89 ± 1.57	2.79 ± 1.40	2.75 ± 1.37	2.80 ± 1.34	2.80 ± 1.45
**Serum 25(OH)D (nmol/L)** ‡	55.4 ± 16.8	53.3 ± 17.2	50.5 ± 16.3	52.2 ± 16.8	50.3 ± 15.6	46.9 ± 15.1††††

The Tromsø study.

^†^ P < 0.01 vs 1s/1s;

^††^ P < 0.05 vs 1f/1f and 2/2;

^†††^ P <0.05 vs 2/2;

^††††^ P < 0.001 vs 1s/1s and 1f/1f; (ANOVA with Bonferroni correction for multiple analyses); Data are number (%) or mean ± SD

*Analysed in 10337 subjects not using BP medication;

**analysed in 6342 subjects;

***analysed in 6702 subjects;

****analysed in 3304 subjects;

‡ analysed in 4501 non-smoking subjects;

When comparing the homozygotes (1s/1s, 1f/1f, 2/2) using ANOVA with Bonferroni correction for multiple analyses, the 1f/1f had significantly lower body height than 1s/1s (P = 0.004); 1s/1s had significantly lower serum calcium than 1f/1f (P = 0.002) and 2/2 (P = 0.031); 1f/1f had significantly higher hip circumference and lower waist/hip ratio (HWR) than 2/2 (P < 0.05); and 2/2 significantly lower serum 25(OH)D than 1s/1s and 1f/1f (P < 0.001) (Table 1).

If these significant differences between the homozygote phenotypes were real and not simply due to chance, one would expect that the corresponding heterozygotes would have mean values in between; and that there would be a significant linear trends across the phenotypes. This was the case for height (phenotypes 1f/1f, 1f/1s, 1s/1s; standardized beta (β) 0.035, P < 0.001), serum calcium (phenotypes 1f/1f, 1f/1s, 1s/1s; β- 0.039, P < 0.01; phenotypes 1s/1s, 1s/2, 2/2; β = 0.026, P < 0.05), hip circumference and HWR (phenotypes 1f/1f, 1f/2, 2/2; β = -0.78 (P< 0.01) and β = 0.049 (P< 0.05), respectively), and for serum 25(OH)D (phenotypes 1s/1s, 1s/2, 2/2 and 1f/1f, 1f/2, 2/2; β = -0.131 (P < 0.001) and β = -0.09 (P < 0.001), respectively). Since there were slightly more women in the 1f/1f group than the other two groups, gender was included as a covariate in the above linear regression models. The analyses were also done sex-specific and the results are shown in Table 2. In both genders the statistically significant differences persisted for serum 25(OH)D, in women for height and in men for serum calcium (Table 2). Regarding height in women, the difference between the 1f/1f and the two other homozygote groups were seen both in women above and below the age of 50 (data not shown).

**Table 2:** Body height, serum calcium, hip circumference, and serum 25(OH)D in relation to homozygote phenotypes in the females and males.

		Homozygote phenotypes		
		1s/1s	1f/1f	2/2
**Females**	**N (%)**	1964 (74.6)	296 (11.3)	371 (14.1)
	**Body height (cm)**	162.2 ± 6.7	160.9 ± 6.8†	162.0 ± 6.4
	**Serum calcium (mmol/L)**	2.38 ± 0.11	2.39 ± 0.10	2.39 ± 0.11
	Circumference hip (cm)*	103.6 ± 9.5	105.1 ± 10.5	102.8 ± 7.6
	Waist/hip (ratio)*	0.82 ± 0.06	0.82 ± 0.06	0.82 ± 0.06
	Serum 25(OH)D (nmol/L)**	54.5 ± 16.7	53.5 ± 17.7	45.7 ± 15.8††
**Males**	**N (%)**	1657 (75.4)	214 (9.7)	327 (14.9)
	**Body height (cm)**	175.8 ± 7.1	175.3 ± 7.2	175.3 ± 6.5
	**Serum calcium (mmol/L)**	2.38 ± 0.10†††	2.40 ± 0.10	2.39 ± 0.10
	Circumference hip (cm)***	103.3 ± 6.4	104.1 ± 6.4	103.1 ± 5.6
	**Waist/hip (ratio**	0.92 ± 0.06	0.91 ± 0.06	0.93 ± 0.06
	Serum 25(OH)D (nmol/L)****	56.9 ± 16.8††††	50.1 ± 15.0	48.4 ± 14.1

The Tromsø study.

^†^ P < 0.05 vs 1s/1s and 2/2;

^††^ P < 0.01 vs 1s/1s and 1f/1f;

^†††^ P < 0.05 vs 1f/1f;

^††††^ P < 0.01 vs 1f/1F and 2/2 (ANOVA with Bonferroni correction for multiple analyses). Data are number (%) or mean ± SD

* Analysed in 1316 females;

** analysed in 1797 non-smoking females;

*** analysed in 1302 males;

****analysed in 1342 non-smoking males

### Clinical endpoints

The number of subjects with incident endpoints MI, T2DM, cancer (including the subtypes colo-rectal, lung, breast and prostate cancer) and death are shown in Table 3. Using the same approach as for the continuous variables and comparing the three homozygote phenotypes, they were significantly different regarding total number of cancer (Chi-square test, P = 0.024), but not regarding MI, T2DM or death. Thus, the number of all cancer was remarkably lower in the 1f/1f group than the two other homozygote groups. To explore this further, the 1f/1f, 1s/1s, 2/2 groups, the 1f/1f, 1f/2, 2/2 groups, the 1f/1f, 1f/1s, 1s/1s groups, the 1s/1s, 1s/2, 2/2 groups as well as three allele score groups defined by a “protection” score (0–2) based on number of 1f alleles (assuming a cancer protective effect of the this allele), were compared with Cox regression including gender as covariate. In the analysis of the allele score group, subjects with two 1f alleles had a 23% reduced incidence of cancer as compared to the reference group with no 1f allele (hazard ratio 0.77, 95% CI 0.62–0.95, P = 0.016); within the group with only homozygote DBP phenotypes the 1f/1f homozygote had a 26% reduced risk as compared to the 2/2 (ref) homozygote (hazard ratio 0.74, 95% CI 0.59–0.93, P = 0.008); within the 1f/1f, 1f/1s, 1s/1s groups the DBP phenotype 1f/1f had a 23% reduced risk as compared with the reference phenotype 1s/1s (hazard ratio 0.77, 95% CI 0.69–0.85, P = 0.016); whereas the other comparisons did not show statistically significant differences (Table 4).

**Table 3:** Number of incident endpoints (MI, T2DM, total cancer and death and types of cancer) after Tromsø 4 in 1994 (those with their first endpoint before Tromsø 4 excluded) in relation to DBP phenotype.

Endpoint	DBP phenotype						Endpoint specific cohort
	1s/1s	1s/1f	1s/2	1f/1f	1f/2	2/2	Cases/controls
**MI (%)**	514 (15.0)	350 (15.2)	451 (14.4)	74 (15.3)	169 (16.2)	102 (15.4)	1660/3139
**T2DM (%)**	279 (8.0)	208 (8.7)	265 (8.2)	44 (8.9)	98 (9.2)	64 (9.4)	958/3489
**Total cancer (%)**	766 (22.5)	491 (21.3)	676 (21.9)	87 (18.3)*	235 (22.5)	155 (23.8)	2410/3117
**Dead (%)**	1330 (36.7)	906 (36.9)	1218 (36.7)	181 (35.5)	424 (38.4)	259 (37.1)	4318/2096
**Colo-rectal cancer (%)**	127 (3.5)	66 (2.7)	128 (3.9)	16 (3.1)	45 (4.1)	37 (5.3)	419/3897
**Lung cancer (%)**	104 (2.9)	75 (3.1)	104 (3.1)	13 (2.6)	31 (2.8)	27 (3.9)	354/3963
**Breast cancer (%)**	87 (4.5)	64 (4.8)	85 (4.6)	17 (5.9)	34 (5.8)	13 (3.6)	300/2218
**Prostate cancer (%)**	118 (7.2)	57 (5.2)	108 (7.5)	13 (6.2)	32 (6.3)	25 (7.7)	353/1625

The Tromsø study.

* P < 0.05 vs 1s/1s and 2/2, Chi-square test

**Table 4:** Gender adjusted Cox hazard ratios and 95% confidence intervals for incident cancer (all locations) according to DBP phenotype groups.

	DBP phenotypes	N	HR	95% CI
**Allele score 0–2**	0 (1s/1s, 1s/2, 2/2)	3588	ref	
	1 (1f/1s, 1f/2)	1697	0.950	0.870–1.037
	2 (1f/1f)	242	0.767 (P = 0.016)	0.618–0.951
**1f/1f, 1f/1s, 1s/1s**	1s/1s	1698	ref	
	1f/1s	1146	0.911	0.813–1.020
	1f/1f	242	0.766 (P = 0.016)	0.688–0.852
**1f/1f, 1f/2, 2/2**	2/2	342	ref	
	1f/2	551	0.986	0.805–1.208
	1f/1f	242	0.782	0.601–1.017
**1s/1s, 1s/2, 2/2**	1s/1s	1698	ref	
	1s/2	1548	0.934	0.846–1.041
	2/2	342	0.939	0.790–1.116
**2/2, 1s/1s, 1f/1f**	2/2	342	ref	
	1s/1s	1698	0.942	0.792–1.119
	1f/1f	242	0.741 (P = 0.008)	0.593–0.925

The Tromsø study.

## Discussion

In the present study we have found significant relations between DBP phenotype and body height, hip circumference, serum calcium, serum 25(OH)D and incidence of cancer, whereas no significant associations were found with MI, T2DM, death, and the CVD risk factors body mass index (BMI), lipids, blood pressure and HbA_1c_.

We measured total and not ionized or albumin adjusted calcium, thus, the association with serum calcium could simply be due to co-variation with serum albumin, since the genes for DBP and albumin are close together [22]. The association with height was found in females only, with a difference of ∼ 1 cm between those with the 1f/1f phenotype and the other two homozygote phenotypes. This difference was seen in both young and older women, and is a considerable height difference as the effect size of individual SNPs associated with height ranges from 0.2 to 0.5 cm per allele in most analyses [23]. Given the association between vitamin D and skeletal health this effect is biologically plausible and, if verified in other studies, could give important biological information since body height has been associated with several cancers [24], CVD [25], as well as mortality [26].

The relation with serum 25(OH)D was as described by others [27] with mean levels for the DBP phenotypes ranging from 46.9 to 55.4 nmol/L. However, there were no relations between the DBP phenotypes and serum PTH which is a fairly sensitive parameter of vitamin Ds biological action. Neither was there any relation with risk factors associated with serum 25(OH)D like BMI, blood pressure, lipids and HbA_1c_, possibly because these relations are not causal, or the differences in serum 25(OH)D levels between the DBP phenotypes were too small to have clinical importance, or were compensated by corresponding differences in binding coefficients for 25(OH)D. Thus, the 1s/1s and 1f/1f phenotypes, which had the highest serum 25(OH)D levels, have binding coefficients for 25(OH)D of 6 x 10^8^ and 11.2 x 10^8^, respectively, whereas the phenotype 2/2, which has the lowest mean serum 25(OH)D level, has a binding coefficient of only 3.6 x 10^8^ [5]. Accordingly, the free fraction of 25(OH)D, which has been suggested to be the biologically active one [4], may not differ much between the DBP phenotypes.

The most important finding in the study was the association between DBP phenotypes and cancer, where the 1f variant appeared to have a cancer protective effect. This was shown both when using an allele score, when comparing the three homozygote phenotypes, and when comparing 1f/1f, 1s/1s and their corresponding heterozygote phenotype. The effect was considerable with a ∼ 25% reduction in cancer incidence related to the 1f allele.

In theory, this relation could be mediated by the effect of DBP on vitamin D metabolism since there are a number of indications that vitamin D may play a role in carcinogenesis; both from observational studies where higher serum 25(OH)D levels are associated with lower incidence of cancer as well as a better prognosis once diagnosed [28]; from in-vitro experiments where 1,25(OH)_2_D modulates malignant cell proliferation [29]; and recently even from a Mendelian randomization analysis where subjects with SNPs associated with low serum 25(OH)D levels had higher cancer mortality [30]. However, an effect on cancer prevention by vitamin D has so far not convincingly been shown in intervention studies [31], and a causal link between vitamin D deficiency and cancer still remains to be proven. Furthermore, since the 1f/1f phenotype had the highest binding coefficient for 25(OH)D, 1f/1f was probably not the phenotype with the highest levels of free or bioavailable vitamin D metabolites. However, vitamin D metabolites do not only enter the cells in their free form. In the proximal tubule cells there are endocytic receptors, megalin and cubilin, that reabsorb the DBP-vitamin D complex for subsequent intracellular conversion of 25(OH)D to 1,25(OH)_2_D [32]. In theory the efficacy of this absorption could depend on DBP phenotype, and if these or similar receptor are present in other than tubule cells [33], this could explain differences related to DBP phenotypes.

An alternative explanation for the relation to cancer could be the macrophage-activating effect of partially deglycosylated DBP (DBP-MAF) [34, 35]. This compound may inhibit proliferation of cancer cells as was shown in a study by Thyer et al. where DBP-MAF-stimulated macrophages were found to attack human breast cancer cells, induce their apoptosis and eventually phagocytize them [36]. And furthermore, and highly relevant to our study, Nagasawa et al. have found the activity of DBP-MAF to be highest in the 1f/1f phenotype [14], the phenotype that in our study appeared to be cancer protective.

The association between DBP phenotypes (or single SNPs in the *GC* gene) and cancer has recently been reviewed by Speeckaert et al. and by Malik et al. [15, 16]. Although several significant associations have been reported, there appears to be no consistent pattern as illustrated by the elevated risk of gastrointestinal cancer associated with the 2/2 phenotype [37], whereas this phenotype appeared to have a protective effect regarding breast cancer [38].

In addition to cancer we also evaluated the relation between DBP phenotypes and MI, T2DM and death, without finding any significant associations. Although such relations cannot be ruled out, it is, considering the high number of subjects with endpoints in our study, highly unlikely that clinically important relations were missed.

Our study has several weaknesses. We did not measure the serum DBP levels, which have been reported to differ in relation to DBP phenotype [39], nor did we evaluate the effect of DBP phenotypes in combination with other genetic or environmental factors, which might have disclosed additional effects [40]. We did not measure the free fraction of serum 25(OH)D which could have been of importance for evaluating cancer risk and vitamin D, nor could we calculate this free fraction as we did not measure serum DBP. Our study also has strengths; we had a large study cohort and had carefully collected and verified clinical endpoints. And most important, our finding of a cancer protective effect of the phenotype 1f/1f could have a plausible biological explanation by the effect on macrophage activation by the DBP-MAF compound.

In conclusion, we have found the DBP phenotype 1f/1f to be associated with a reduced risk of cancer. If this is confirmed in other studies, determination of DBP phenotype could prove to be of clinical importance.
